# Treatment of Danhong Injection Combined with Naoxintong Capsule in Acute Coronary Syndrome Patients Undergoing PCI Operation: Study for a Randomized Controlled and Double-Blind Trial

**DOI:** 10.1155/2018/8485472

**Published:** 2018-03-07

**Authors:** Shuai Zhao, Yong Tang, Hairong Cai, Weifeng Liu, Lieyuan Zhang, Dongjie Chen, Bojun Chen

**Affiliations:** ^1^Second Clinical College of Guangzhou University of Chinese Medicine, Guangzhou 510120, China; ^2^Third Affiliated Hospital of Guangzhou University of Chinese Medicine, Guangzhou 510378, China; ^3^Foshan Hospital of Traditional Chinese Medicine, Foshan 528000, China; ^4^Guangdong Province Hospital of Integrated Traditional Chinese and Western Medicine, Foshan 528200, China

## Abstract

**Objective:**

This double-blind and randomized placebo-controlled trial evaluated the safety and efficacy of Danhong injection combined with Naoxintong capsule in patients with acute coronary syndrome (ACS) after percutaneous coronary intervention (PCI).

**Methods:**

ACS patients scheduled to undergo PCI (*n* = 130) were equally and randomly apportioned to either a treatment or control group. After PCI, the treatment group received Danhong injection combined with Naoxintong capsule for 12 weeks, while the control group was given placebo. Both groups were otherwise treated with conventional secondary prevention of coronary artery disease. The groups were primarily evaluated for clinical efficacy and cardiovascular events. Evaluative indicators of safety included adverse events, platelet count, and liver, renal, and blood coagulation functions.

**Result:**

No cardiovascular events or adverse reactions were observed in either group. The treatment group demonstrated better signs of clinical efficacy, including left ventricular ejection fraction, higher nitric oxide levels, and lower levels of endothelin-1 (ET-1) and von Willebrand factor (VWF).

**Conclusion:**

ACS patients treated with Danhong injection combined with Naoxintong capsule after PCI demonstrated better improvement with regard to markers associated with atherosclerosis and adverse cardiovascular events, without apparent adverse effects. Thus, Danhong injection combined with Naoxintong capsule was safe and effective for treating ACS patients after PCI.

## 1. Introduction

Acute coronary syndrome (ACS) generally refers to a sudden decrease in blood flow to the coronary arteries, or acute myocardial ischemia, including ST segment elevation myocardial infarction (STEMI), non-STEMI (NSTEMI), and unstable angina. ACS is a worldwide health issue with high morbidity and mortality [1] and is the second leading cause of death in coronary heart disease. In China, rates of hospitalization and hospital mortality due to ACS have been rising and the disease is now a leading cause of death and disability [2].

The mainstay treatment for ACS is percutaneous coronary intervention (PCI), which can quickly reconstitute the blood supply and effectively remove vascular stenosis or occlusion [3]. However, PCI cannot prevent the progression of atherosclerosis. Rather, PCI has the potential to promote the formation of early acute or subacute small blood clots and activate platelets, subsequently leading to stent restenosis [4].

Indeed, it is widely believed that vascular endothelial dysfunction is the initiating factor for atherosclerosis [5], and stent restenosis has been linked to injury of vascular endothelial cells in the coronary artery intima during PCI [6]. In addition, PCI-induced vascular endothelial cell dysfunction was found to promote plaque rupture and thrombosis, leading to the conclusion that this was the main cause of stent restenosis after PCI.

Markers of endothelial function include plasma nitric oxide (NO), endothelin-1 (ET-1), and von Willebrand factor (VWF). NO is an indicator of vasodilatation [7], and ET-1 is a vasoconstrictor [8]. Elevated plasma levels of VWF have been linked to thrombosis [9]. Abnormal levels of NO, ET-1, and VWF promote endothelial dysfunction that has been associated with atherosclerosis and as such are important indicators in vascular biology and pathophysiology. Indeed, levels of circulating NO, ET-1, and VWF are independent predictors of coronary thrombosis, stent restenosis, and other adverse cardiovascular events [10].

Danhong injection is a traditional Chinese medicine (TCM) that promotes blood circulation to remove blood stasis and regulate body function [11]. Its main components are Tanshinone and* Salvia miltiorrhiza* safflower yellow pigment,* Salvia miltiorrhiza* phenolic acid, safflower phenolic glycosides, and catechol. Studies have shown that Danhong injection can prevent atherosclerosis, ameliorate oxidative stress, attenuate inflammatory injury, regulate blood lipids, balance blood pressure, and reduce blood viscosity [12]. In addition, Danhong injection was shown to significantly reduce myocardial cell degeneration and necrosis caused by ischemia, alleviate the onset of angina pectoris, and improve myocardial blood perfusion [12].

The major components of Naoxintong capsule are milkvetch root, leech, earthworm, scorpion,* Salvia miltiorrhiza*,* Angelica sinensis*, Radix Paeoniae Rubra, Rhizoma Ligustici wallichii, peach kernel, safflower, frankincense, myrrh, Caulis Spatholobi, ramulus mori, achyranthes, and cassia twig. Naoxintong capsule supplements qi, that is, the basic material that makes up the human body and blood circulation according to the theory of TCM [13]. Previous studies reported that Naoxintong capsule protected myocardial and endothelial cells, promoted coronary collateral circulation [14], and reduced the risk of cardiovascular disease morbidity.

Danhong injection and Naoxintong capsule have been widely used in a variety of cardiovascular and cerebrovascular diseases. However, no randomized controlled trials have been performed to evaluate the safety and efficacy of the combined use of Danhong injection and Naoxintong capsule for treatment of ACS patients after PCI.

The present study was a multicenter, randomized, double-blind, placebo-controlled, and parallel design clinical trial that investigated the safety and efficacy of the combined use of Danhong injection and Naoxintong capsule in patients with ACS after PCI. We explored the molecular basis underlying the beneficial effects of the combined use of these two Chinese traditional medicines in these patients.

### 1.1. Ethics

The Ethics Committee at Guangdong Hospital of Traditional Chinese Medicine reviewed and approved the study. All patients signed informed consent before the study, and the study was conducted in accordance with the principles of clinical practice and Helsinki declaration. The trial was registered in the China Clinical Trial Registration Center (registration number: ChiCTR-IOR-14005693). The test protocol was approved by the Ethics Committee of Guangdong Provincial Hospital of Traditional Chinese Medicine (ethical batch number: B2014-031-01).

## 2. Methods

### 2.1. Trial Design

This study was a multicentered, randomized, double-blind, placebo-controlled, and parallel-designed clinical trial involving ACS patients after PCI. All patients who met the inclusion criteria were randomly allocated to the control group and the treatment group according to the ratio of 1 : 1 based on the random numbers that were computer-generated by researchers who did not work for the research institutions (i.e., the clinical research methodology team at Guangdong Hospital of TCM). The numbers were the same as those shown on the medication package and the drug package used in the study. The study was double blinded; that is, patients and medical staff were blinded to the treatment and placebo groups, but not the data managers. Only the data manager in each center knew which group a patient was assigned to.

### 2.2. Participants

**Figure 1:**
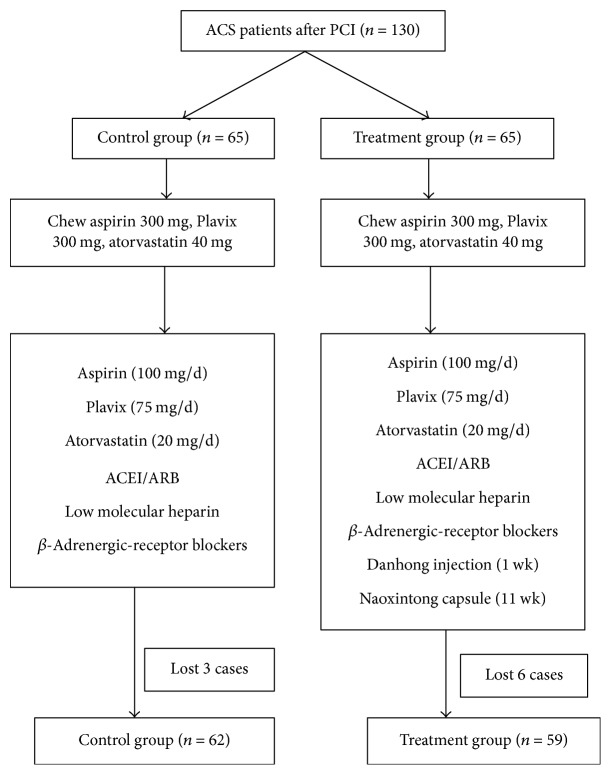
Subject selection and treatments. ACEI: angiotensin converting enzyme inhibitor; ARB: angiotensin II receptor antagonist.

ACS patients scheduled to undergo PCI (*n* = 130) from September 2014 to December 2015 were selected for this study. Eighty of the patients were treated at Second Affiliated Hospital of Guangzhou University of Chinese Medicine (Guangdong Province), and 50 were at Maoming City People's Hospital. Patients were apportioned to a treatment group or a control group according to a random number table (Figure 1).

#### 2.2.1. Inclusion and Exclusion Criteria

Patients who met the following conditions were included in this study: a diagnosis of ACS (see below); age: 50–80 y; and informed consent signed by self or relatives. Patients with any of the following were excluded from our study: allergy to Danhong injection or Naoxintong capsule; no inspection record to evaluate safety and efficacy indicators; or use of any drug during the study that would affect the evaluation of clinical effects or safety. In addition, patients with any of the following diseases or complications were excluded: serious cardiac arrhythmias (e.g., type II atrioventricular block or sick sinus syndrome); severe hypotension; hypertension emergency; severe heart failure (cardiac function > III not effectively controlled); acute cardiac tamponade; acute pulmonary edema; hemorrhagic cerebrovascular diseases; gastrointestinal hemorrhage; hemophilia; severe thrombocytopenia; coagulation disorder; serious diabetic complications (diabetic ketoacidosis); severe liver or kidney impairment (serum alanine aminotransferase [ALT] > 3-fold normal ceiling or serum creatinine ≥ 265 *μ*mol/L); malignant tumor; psychosis; or coronary artery bypass grafting surgery (i.e., heart bypass surgery).

Participants were considered lost during the study if the researchers determined that the subject should discontinue due to aspartic acid transaminase (AST) or ALT > 3-fold the upper limit of the normal range in 2 consecutive examinations; creatinine > 1.5-fold the upper limit of normal in 2 consecutive examinations; severe adverse reactions; other diseases which influenced the curative effects or evaluation of adverse events; the participant being not suitable for continuing use of the study drug; or the patient becoming unblinded to the treatment. In addition, patients were dropped from the study if they discontinued participation by withdrawing informed consent, were unwilling to continue because of poor effects or symptomatic deterioration, could not tolerate the adverse events, or died.

ACS was diagnosed based on the recommended criteria of the American College of Cardiology/American Heart Association with regard to STEMI and NSTEMI-ACS. Characteristics of STEMI included symptoms associated with myocardial ischemia, ST segment elevation on electrocardiogram (ECG), and a change in myocardial damage markers (troponin I, troponin T, and creatine kinase isoenzyme). Blood samples for the detection of myocardial damage markers were collected ≥ 2x within an interval of 6 hours.

Features of NSTEMI-ACS were apparent within one month of new angina, or original angina progression within one month or resting state angina, accompanied by a deelevation of the ST segment on ECG. NSTEMI was diagnosed if the concentrations of the myocardial damage biomarkers (troponin I, troponin T, and creatine kinase isoenzyme) were above the normal range. Otherwise unstable angina was considered if the myocardial damage biomarkers were within normal.

#### 2.2.2. Sample Size Determination

According to [15], the effective rate of secondary prevention of coronary heart disease in patients with ACS in China is 76%, and the effective rate of secondary prevention of coronary heart disease combined with Danhong injection is 86%. Assuming *δ* = 0.08, *α* = 0.05, and *β* = 0.20 and using a noninferiority test design (i.e., the treatment group after 3 months has a higher efficiency than the control group), the following formula was used to determine the sample size:

(1) n1=n2=Z1−α/2+Z1−β2π11−π1+π21−π2π1−π2−δ2.

Thus, the number of samples should be at least 54 in each group. Considering a 20% loss rate, it was estimated that there should be a minimum of 65 cases in each group and at least 130 cases overall.

#### 2.2.3. Randomization

A hierarchical stochastic allocation scheme was used for randomization, in which the stratification factor was 2 centers. Each center adopted the method of block randomization to set the block size to 4, and the patients who met the inclusion criteria were randomly allocated to the control group and the treatment group according to a 1 : 1 ratio. A randomized, multicenter trial was performed by nonparticipating research institute researchers (from the Clinical Research Methodology Group of Chinese Medicine and Guangdong Provincial Hospital of Traditional Chinese Medicine). The numbers were the same as those shown on the medication package and the drug package used in the study.

### 2.3. Intervention

Danhong injection (specification: 10 mL/branch; drug batch number: 14081017) and Naoxintong capsule (drug batch number 141080) were provided by Shaanxi Buchang Pharmaceutical. The placebo injection consisted of 0.9% sodium chloride. The Danhong injection and placebo injections were blinded by shading the intravenous infusion. The placebo capsules to replace Naoxintong capsules in the control group contained excipients and were provided by Shaanxi Buchang Pharmaceutical. The Naoxintong capsules and placebo capsules were identical in appearance, weight, packaging, specifications, and fonts.

### 2.4. Drug Delivery Methods

All patients who met the inclusion criteria were given aspirin 300 mg, Plavix 300 mg, and atorvastatin calcium tablet 40 mg (chewing) after admission. For the patients with STEMI, PCI after thrombolysis or selective PCI was chosen according to the time interval from the onset of chest pain to admission and hemodynamic factors. For the unstable angina/NSTEMI patients, high-risk patients underwent PCI treatment as soon as possible, while low-risk patients had elective PCI therapy.

Eventually, all patients who underwent PCI surgery were given secondary prevention of coronary artery disease after surgery, including aspirin (100 mg/d), Plavix (75 mg/d), atorvastatin calcium tablet (20 mg/d), angiotensin converting enzyme inhibitors (ACEIs), angiotensin II receptor antagonists (ARBs), and low molecular heparin. Those patients with no contraindications were given *β*-adrenergic-receptor blockers.

Patients in the treatment group were given Danhong intravenous injection (20 mL Danhong added in 5% glucose 100 mL/0.9% sodium chloride, 100 mL, gtt, qd, 1x/wk) for one week and then Naoxintong capsule (4x, po, tid, 11 wk) for 11 weeks. The control group was given a placebo injection and placebo capsules as follows: placebo injection, 20 mL sodium chloride injection added in 5% glucose injection 100 mL/0.9% sodium chloride injection 100 mL iv, gtt, qd × 1 week; and placebo capsule, starch preparation, 4/time, po, tid × 11 week. The total treatment lasted 12 weeks.

The end point or an early termination point of the trail was considered the completion of observation, including the baseline and double-blinded treatment period of 1 week and 12 weeks for a total of 3 times. Patients who discontinued were considered lost to the study.

The following were recorded upon hospitalization on the first day, the first week, and the second week: number of chest pains, routine blood examination, liver function, renal function, blood coagulation, ECG, levels of NO, ET-1, VWF, adverse reactions, and cardiovascular events.

### 2.5. Outcomes

The primary outcomes were clinical curative effects and cardiovascular events. Clinical curative effects were rated excellent, effective, or ineffective, based on the clinical efficacy criteria recommended by the Drug Administration of the National Ministry of Health. A curative effect was considered excellent when symptoms disappeared or improved significantly, the frequency of chest pain attacks was reduced by 80%, and ECG returned to normal or roughly normal. Results of treatment were defined as effective if symptoms improved; chest pain attacks were reduced by 50–80%; ECG showed a decreased ST segment ≥ 0.05 mV, ST segment fell ≥ 0.05 mV but did not return to normal, T wave in the primary lead changed from flat to upright, or >50% of the T wave inversion was lighter. The treatment was considered ineffective when either there was no change in clinical symptoms or symptoms aggravated; chest pain attacks showed no significant change; and the ST segment on ECG showed no change or aggravated.

Cardiovascular events within 3 months were recorded as follows: nonfatal myocardial infarction; vascular blood supply and revascularization; hospitalization because of severe angina or heart failure; or resulting in mortality. Adverse cardiovascular events included cardiac death, cardiac shock, arrhythmia, repeat myocardial infarction, revascularization, readmission due to ACS, recurrent myocardial ischemia, cardiac insufficiency, or nonfatal stroke. In addition, general adverse events or reactions were observed. Since this study used listed drugs, the adverse events that occurred during this trial were mainly dizziness, anxiety, and stomach pain. Adverse events or reactions, as well as new disease or aggravation of original symptoms, were recorded. Serious adverse events were reported according to the requirements of good clinical practice.

The secondary outcomes included parameters from laboratory examinations: plasma ET-1, NO, VWF, and cardiac function such as left ventricular ejection fraction (LVEF) through echocardiography. Safety observation indexes included routine blood exam and liver, renal, and coagulation functions.

Regular laboratory routine tests included blood, urine, stool, liver and renal functions, 4 items of blood lipids, and fasting blood-glucose. For the endothelial function index, 6 mL of blood was taken from the elbow vein of all patients (fasting) and centrifuged. The separated plasma was immediately frozen at −450°C. The levels of circulating ET-1, NO, and VWF were measured at the King Med Diagnostics Center.

### 2.6. Statistical Analysis

According to the intention-to-treatment population analysis principle, the last observation carried forward method was used for analysis of clinical efficacy to deal with missing data and information dropout. Baseline data of the study were computed by corresponding appropriate statistical analyses based on the nature of the data, that is, the mean for continuous variables, and frequency distribution for classification variables. Clinical efficacy was analyzed by comparing changes in the evaluation indexes in the respective individuals of the 2 groups from baseline to each observation period. All analyses were performed using a 2-sided test, and *P* < 0.05 was considered statistically significant.

Measurement data are described as the number of cases, mean ± standard deviation, minimum, maximum, and the median and were statistically analyzed using a *t*-test, analysis of variance, or Wilcoxon rank sum test. Various count data were described as case number and percentage and were statistically analyzed using the chi-squared or Wilcoxon rank sum tests. Ranked data are described as the number of cases and percentage and statistically analyzed using the Wilcoxon rank sum test.

## 3. Results

### 3.1. Demographic and Basal Clinical Characteristics

A total of 130 ACS patients, all of whom had either NSTEMI or STEMI and were scheduled for PCI, were enrolled in the present study (Table 1; Figure 1). The 130 patients were equally and randomly assigned to receive either treatment consisting of Danhong injection and Naoxintong capsule (treatment group; 36 men and 29 women; aged 68.88 ± 5.75 years) or a placebo injection and placebo capsule (control group; 38 men and 27 women; aged 67.26 ± 5.49 years). There were no statistically significant differences between the treatment and control groups with regard to the following demographic and basic clinical characteristics: age, gender ratio, blood pressure, body mass index, distribution of risk factors of coronary heart disease, and cardiac function classification according to the New York Heart Academy.

### 3.2. Clinical Efficacy

During a 12-week follow-up period, 6 and 3 cases were lost in the treatment and control groups, respectively, and these patients were removed based on intention-to-treat. In the twelfth week of follow-up, for the treatment group the clinical curative effects were judged as excellent, effective, and ineffective in 35, 16, and 8 patients, respectively (Table 2). In the control group, the curative effects were rated excellent, effective, and ineffective in 27, 17, and 18 patients. The overall response rate of the treatment group (86.44%) was significantly higher than that of the control group (70.96%).

### 3.3. Cardiovascular Events

During the 12-week treatment period, no patient in either group experienced any serious event such as death, nonfatal myocardial infarction, target blood supply vessel revascularization, recurrence of myocardial infarction, hospitalization for recurrent myocardial ischemia or severe heart failure, or severe angina events.

### 3.4. Secondary Indexes

#### 3.4.1. Circulating NO

Prior to treatment, the mean levels of NO of the treatment and control groups were similar (*P* > 0.05; Table 3). At 7 days of treatment the levels of NO had increased in both groups, but the change in the treatment group was significantly higher than that of the control group. At 12 weeks of treatment the level of NO had significantly elevated in both groups, but the change in the treatment group was obviously larger than that in the control group (*P* < 0.05).

Therefore, we conclude that combined use of Danhong injection and Naoxintong capsule substantially increases the circulating level of NO in ACS patients undergoing PCI compared with the control group.

#### 3.4.2. Circulating ET-1 and VWF

Prior to treatment, the mean levels of ET-1 and VWF of the treatment and control groups were similar (*P* > 0.05; Table 3). At 7 days of treatment, the levels of ET-1 and VWF had decreased in both groups, but the change in the treatment group from baseline was less than that of the control group. At 12 weeks of treatment, the ET-1 and VWF levels of both groups were significantly lower than at the baseline, but the decrease in the treatment group was significantly less than that of the control. Hence, we conclude that the combined use of Danhong injection and Naoxintong capsule sustains ET-1 and VWF levels better than the placebo in ACS patients after PCI.

#### 3.4.3. LVEF

Prior to treatment, the mean LVEF of the treatment and control groups were statistically similar (Table 4). At 7 days of treatment, the LVEF had increased in both groups, but the LVEF of the treatment group was significantly higher than that of the control group. It was surprising that, at 12 weeks of treatment, the LVEF of both groups were less than those at 7 weeks, but the LVEF remained significantly higher in the treatment group than the control group.

Collectively, the combined use of Danhong injection and Naoxintong capsule improved cardiac function better than the placebo in ACS patients undergoing PCI.

### 3.5. Evaluation of Safety

Based on the principle of intention-to-treat, the last observation carried forward approach was used to deal with missing data (Table 5). No significant differences in the levels of PLT, AST, ALT, BUN, CR, and INR were observed between the treatment and control groups at baseline, and after treatment these levels had not changed from baseline. This indicated that neither the combined use of Danhong injection and Naoxintong capsule nor secondary coronary heart disease prevention had any significant influence on the functions of major organs.

### 3.6. Adverse Reactions

At no time during the study period did any serious adverse events occur in either the treatment or control group. Furthermore, none of the following adverse reactions was noted in any of the patients of either group: headache, dizziness, palpitation, chills, fever, facial blushing, nausea, vomiting, diarrhea, or chest distress.

## 4. Discussion

We employed a randomized, double-blind study design to explore the clinical benefits of the combined use of Danhong injection and Naoxintong capsule for ACS patients after PCI. Compared to the control patients, we found that the combination of these traditional Chinese medicines was associated with clinical benefits and improved endothelial function, without detectable cardiovascular events or other adverse reactions.

ACS is a clinical syndrome caused by myocardial ischemia due to thrombosis. Myocardial reperfusion therapy is the most direct and effective treatment for STEMI and NSTEMI, and PCI is recognized as the mainstay means to achieve blood reperfusion in the shortest time [3]. According to China's PCI guide (2009) for treatment of unstable angina and NSTEMI, the selection of PCI therapy should be based on risk stratifications and is not recommended for patients who are at low-risk

While there has been rapid progress in PCI technology for the treatment of ACS, some clinical issues after PCI remain, such as promoting the formation of early acute and subacute small blood clots, activating platelets leading to stent restenosis, promoting antiplatelet drug resistance, and leading to poor myocardial reperfusion [5]. Some clinical studies have suggested that the use of TCM alone, or TCM integrated with Western medicine, for the treatment of ACS can offer significant benefits to patients [11]. For example, Danhong injection was shown to promote blood circulation under conditions of blood stasis, regulate the body's physiology, and activate antiplatelet aggregation, anti-inflammation, antiapoptosis, and vascular endothelium protection [16]. Naoxintong capsule was shown to supplement qi, activate blood circulation, and eliminate hemostasis and free channels, and it also possessed antioxidant, antihyperlipidemic, antiatherosclerosis, and vasodilation properties [17]. Thus, Danhong injection and Naoxintong capsule have been widely used in a variety of cardiovascular and cerebrovascular diseases.

In addition, we did not observe any associated adverse effects, although rare adverse reactions linked to Danhong injection have been reported [18]. Therefore, we believed that the combined use of these two medicines was safe and effective for treatment of ACS patients after PCI.

Vascular endothelial cells, as the largest autocrine, paracrine, and endocrine organs in humans, regulate the dynamic balance of environment for blood vessel walls, both inside and outside, mainly by compounding and releasing the vascular active factor. NO and ET-1 are the two main vascular active factors, while NO influences vasodilatation, antiplatelet aggregation, antithrombosis, and inhibition of excessive proliferation of smooth muscle cells [8, 19]. VWF is a glycoprotein synthesized and secreted by vascular endothelial cells and macrophages that can cause blood vessels to contract [20]. Therefore, the balance between NO and ET-1/VWF is important for the homeostasis of vascular endothelial cells. An increase in ET-1/VWF and decrease in NO can cause vasospasm, smooth muscle cell proliferation, blood clots, and the hardening of plaques [21]. Hence, NO, ET-1, and VWF are considered sensitive indexes of endothelial cell injury and risk factors for thrombosis, severity of morbidity, and prognosis. In the present study, we found that Danhong injection combined with Naoxintong capsule was associated with significantly greater NO levels and lower plasma levels of ET-1 and VWF compared with the control group, which probably contributed to the benefits of these medications for ACS patients after PCI.

Previous studies have shown that ACS is not completely dependent on the degree of coronary narrowness but rather is closely related to endothelial dysfunction [22, 23]. Vascular endothelial dysfunction can lead to a reduction in NO and promote platelet aggregation and infiltration of inflammatory factors, which predisposes an unstable plaque to rupture, subsequently leading to ACS [24, 25]. Therefore, although PCI is the mainstay treatment for ACS, it also causes clinical side effects including stent restenosis.

Two major mechanisms contribute to PCI-linked restenosis. Firstly, PCI causes a decrease in NO release and increases in ET-1 and VWF [26, 27], promoting the release of angiotensin and stent restenosis. Secondly, PCI causes vascular endothelial cell injury, a subsequent decrease in anticoagulation substances, and the formation of subacute and late thrombosis [28]. Thus, vascular endothelial cell dysfunction is well recognized as the main mechanism leading to PCI-linked stent restenosis [29, 30]. Conversely, improving endothelial cell function was found to prevent stent restenosis after PCI [31]. In the present study, the combined use of Danhong injection and Naoxintong capsule ameliorated changes in the levels of circulating NO, ET-1, and VWF in our patients and, therefore, endothelial function as well.

### 4.1. Limitations

Although we obtained positive results in the present study, we did not examine end points such as all-cause mortality, and a follow-up time of 3 months is relatively short. In addition, the sample size was relatively small although it reached requirement for power analysis. Thus, a multicenter study with a large cohort should be performed to corroborate our observations.

## 5. Conclusion

Danhong applied as an injection is not appropriate for long-term use mainly due to the following: First, as an injection for intravenous infusion, the administration of Danhong is inconvenient for long-term use. Second, Danhong injection promotes blood circulation and is a collateral analgesic, which is only suitable for short-term use during the acute phase; long-term use will increase qi, which is not appropriate. ACS within 1 week is considered the acute phase, so Danhong injection should be used during this period. However, in the clinical setting Danhong injection is practical when combined with Naoxintong capsule. This conforms to the principle of Chinese medicine: to treat acute conditions symptomatically and chronic conditions radically. From the present study, we conclude that the combined application of Danhong injection and Naoxintong capsule can benefit ACS patients after PCI, compared with Western medications alone, by improving endothelial function. Furthermore, the treatment did not cause any detectable adverse reactions. Therefore, Danhong injection combined with Naoxintong capsule for treatment of ACS patients undergoing PCI is safe and effective.

## Figures and Tables

**Table 1:** Demographic and basic clinical characteristics of treatment and control groups^*∗*^.

	Treatment	Control
Initial subjects, *n*	65	65
Age, year	68.88 ± 5.75	67.26 ± 5.49
Gender, *n*, male/female	50/40	53/37
Systolic pressure, mmHg	148.79 ± 13.01	147.74 ± 11.83
Diastolic pressure, mmHg	77.76 ± 6.76	76.79 ± 6.55
Body mass index, kg/m^2^	23.20 ± 1.63	23.05 ± 1.65
Risk factor, *n* (%)		
Smoking history	41 (45.6)	38 (42.2)
Drinking history	35 (38.9)	32 (35.6)
Hypertension	60 (66.7)	62 (68.9)
Hyperlipidemia	36 (40.0)	41 (45.6)
Diabetes mellitus	23 (25.6)	22 (24.4)
Cardiac function 1/2/3/4, *n*	8/49/33/0	11/46/33/0

^*∗*^Reported as mean ± standard deviation, unless noted otherwise.

**Table 2:** Clinical efficacy ratings of the treatment and control groups.

	Subjects, *n*	Excellent	Effective	Ineffective	Overall response, %	*Z*	*P*
Treatment	59	35	16	8	86.44	−2.068	0.039
Control	62	27	17	18	70.96		

*Notes.* Analyzed by rank sum test.

**Table 3:** Comparison of secondary outcomes between the treatment and control groups.

	Treatment	Control	*t*	*P*
Subjects, *n*	65	65		
NO, *μ*mol/L				
Baseline	52.57 ± 3.27	53.10 ± 2.84	−0.978	0.330
Day 7	62.79 ± 3.04	60.71 ± 2.53	4.225	≤0.001
Week 12	73.86 ± 4.37	69.12 ± 2.52	7.581	≤0.001
ET-1, ng/L				
Baseline	124.26 ± 3.56	124.71 ± 3.37	−0.744	0.458
Day 7	81.93 ± 1.63	87.48 ± 1.29	−21.421	≤0.001
Week 12	67.52 ± 4.85	76.17 ± 3.10	−12.104	≤0.001
VWF, ng/mL				
Baseline	207.55 ± 7.77	207.06 ± 7.64	0.363	0.717
Day 7	192.33 ± 3.79	195.69 ± 3.79	−5.041	≤0.001
Week 12	179.89 ± 5.09	185.82 ± 3.52	−7.714	≤0.001

Equal variance, analyzed by Levene's test for equality of variances (*P* > 0.05); *t* and *P* values were, respectively, statistics and probability which were analyzed by paired *t*-test for the two groups before treatment.

**Table 4:** Comparison of secondary outcomes (LVEF) between the treatment and control groups.

	Subjects, *n*	Baseline	Day 7	Week 12
Treatment	65	48.80 ± 4.38	61.24 ± 4.91	70.55 ± 3.94
Control	65	48.00 ± 3.73	56.33 ± 3.96	65.40 ± 3.35
*t*		1.120	6.263	8.016
*P*		0.265	≤0.001	≤0.001

*Notes.* Equal variance, analyzed by Levene's test for equality of variances (*P* > 0.05); *t* and *P* values were, respectively, statistics and probability which were analyzed by paired *t*-test for the two groups before treatment.

**Table 5:** Comparison of PLT, AST, ALT, BUN, CR, and INR in participants of two groups before and after treatment.

	Baseline	After treatment	*t*	*P*
PLT, ×10^9^/L				
Treatment	181.00 ± 32.51	180.95 ± 32.58	0.008	0.994
Control	187.60 ± 41.59	188.98 ± 42.49	−0.188	0.851
AST, U/L				
Treatment	19.53 ± 8.04	19.70 ± 7.05	−0.127	0.899
Control	19.23 ± 6.77	18.69 ± 6.27	0.470	0.639
ALT, U/L				
Treatment	18.01 ± 5.29	17.63 ± 5.65	0.400	0.690
Control	17.18 ± 5.34	16.52 ± 5.24	0.712	0.478
BUN, mmol/L				
Treatment	5.05 ± 1.02	4.91 ± 0.93	0.785	0.434
Control	4.85 ± 0.94	4.84 ± 0.85	0.059	0.953
CR, *μ*mol/L				
Treatment	65.98 ± 9.27	64.76 ± 8.18	0.792	0.430
Control	64.95 ± 8.70	63.61 ± 7.07	0.962	0.338
INR, R				
Treatment	1.01 ± 0.11	1.00 ± 0.09	0.279	0.780
Control	1.00 ± 0.11	0.99 ± 0.08	0.406	0.685

*Notes.* Equal variance, analyzed by Levene's test for equality of variances (*P* > 0.05); *t* and *P* values were, respectively, statistics and probability which were analyzed by paired *t*-test for the two groups before treatment.
